# From Digital Health to Digital Well-being: Systematic Scoping Review

**DOI:** 10.2196/33787

**Published:** 2022-04-04

**Authors:** Merlijn Smits, Chan Mi Kim, Harry van Goor, Geke D S Ludden

**Affiliations:** 1 Department of Surgery Radboud University Medical Center Nijmegen Netherlands; 2 Department of Design, Production, and Management Faculty of Engineering Technology University of Twente Enschede Netherlands

**Keywords:** well-being, design, evaluation, technology assessment, digital health, eHealth, mHealth, telehealth, mobile phone

## Abstract

**Background:**

Digital health refers to the proper use of technology for improving the health and well-being of people and enhancing the care of patients through the intelligent processing of clinical and genetic data. Despite increasing interest in well-being in both health care and technology, there is no clear understanding of what constitutes well-being, which leads to uncertainty in *how* to create well-being through digital health. In an effort to clarify this uncertainty, Brey developed a framework to define problems in technology for well-being using the following four categories: epistemological problem, scope problem, specification problem, and aggregation problem.

**Objective:**

This systematic scoping review aims to gain insights into how to define and address well-being in digital health.

**Methods:**

We followed the PRISMA-ScR (Preferred Reporting Items for Systematic Reviews and Meta-Analyses extension for Scoping Reviews) checklist. Papers were identified from 6 databases and included if they addressed the design or evaluation of digital health and reported the enhancement of patient well-being as their purpose. These papers were divided into design and evaluation papers. We studied how the 4 problems in technology for well-being are considered per paper.

**Results:**

A total of 117 studies were eligible for analysis (n=46, 39.3% design papers and n=71, 60.7% evaluation papers). For the *epistemological problem*, the thematic analysis resulted in various definitions of well-being, which were grouped into the following seven values: *healthy body*, *functional me*, *healthy mind*, *happy me*, *social me*, *self-managing me*, and *external conditions*. Design papers mostly considered well-being as *healthy body* and *self-managing me*, whereas evaluation papers considered the values of *healthy mind* and *happy me*. Users were rarely involved in defining well-being. For the *scope problem*, patients with chronic care needs were commonly considered as the main users. Design papers also regularly involved other users, such as caregivers and relatives. These users were often not involved in evaluation papers. For the *specification problem,* most design and evaluation papers focused on the provision of care support through a digital platform. Design papers used numerous design methods, whereas evaluation papers mostly considered pre-post measurements and randomized controlled trials. For the *aggregation problem*, value conflicts were rarely described.

**Conclusions:**

Current practice has found pragmatic ways of circumventing or dealing with the problems of digital health for well-being. Major differences exist between the design and evaluation of digital health, particularly regarding their conceptualization of well-being and the types of users studied. In addition, we found that current methodologies for designing and evaluating digital health can be improved. For optimal digital health for well-being, multidisciplinary collaborations that move beyond the common dichotomy of design and evaluation are needed.

## Introduction

### Background

Digital health technologies are increasingly being used to monitor, manage, and support health and well-being. The use of digital health promises to increase access to health information, improve the quality of care, reduce errors, and stimulate healthy behavior [1]. Digital health delivery is also often referred to as *eHealth* [2]. Subsets of eHealth are the application of a specific technology in health delivery (eg, mobile health technologies [3]) or the use of digital technology for a specific purpose (eg, remote delivery of health care services: *telehealth* or *telemedicine* [4,5]). The COVID-19 crisis has stimulated the use of digital health, and its application is expected to increase in the coming years [6-8].

The term *digital health* is defined as the “proper use of technology for improving the health and wellbeing of people at individual and population levels, as well as enhancing the care of patients through intelligent processing of clinical and genetic data” [9]. In this paper, we aim to shed light on the term *well-being* in this definition. Studies on the meaning of well-being date back to ancient Greece. Since then, many disciplines have reflected on this term. Well-being is commonly considered as “a state of persons which designates that they are happy or flourishing and that their life is going well for them” [10]. It is seen as “the highest value to which other values can be subsumed” [10]. In this, *values* are considered as everything people consider important in life [11].

Well-being is gaining increasing interest in both health care and technology. In health care, *health* has long been regarded as the absence of disease or infirmity. Its formal definition was changed to *well-being* in 1948. In that year, the World Health Organization redefined health as “a state of complete physical, mental, and social wellbeing” [12]. In technology, well-being is often considered to be a central value in the design process. Many technologies aim to improve the well-being of their users. To that end, an increasing number of design methodologies exist that aim to guide design for well-being processes [10].

However, there is no clear explanation of what (values) constitute well-being, which leads to differences in understanding well-being and much uncertainty on how to enhance well-being through digital health. Philosopher Philip Brey [10] categorizes uncertainty by means of 4 problems that are paramount in technology for well-being. First, he describes the *epistemological problem.* This problem refers to the definition of well-being that should be embedded in technology design. This includes questions on how users consider their own well-being and how designers should obtain an understanding of the conceptions of users. The *scope problem* refers to questions on the *who* and *when* of considering well-being in design. A design can improve the well-being of its main user group but could also include needs from indirect users. In addition, the focus of improving well-being could be short term, long term, or both. The third problem, the *specification problem,* includes all the questions related to the embodiment of well-being in technology design: how should well-being be translated into design requirements, and how can one ensure that users will experience improved well-being when using technology? The fourth problem, the *aggregation problem*, refers to dealing with conflicts that arise between the contradictory values of well-being. Value conflicts can occur in one user (eg, when choices for optimal short-term well-being do not correspond to choices necessary for long-term well-being) or between users of similar or different groups (when an increase in the well-being of one user leads to a decrease in the well-being of another user). The 4 major questions in technology for well-being challenge the ability to create digital health for optimal well-being.

### Objective

In this study, we conducted a systematic scoping review to facilitate future practitioners in the process of creating digital health for the well-being of patients. Digital health commonly follows two processes before adoption takes place: *design* and *evaluation*. Design is the process in which technology is created with the objective of solving a specific problem in health care. Evaluation is the process of (scientifically) assessing the added value of a technology to decide whether to adopt it as part of standard care. We will reflect on the current practices in both design and evaluation to find an answer to how to deal with the 4 problems paramount in technology for well-being.

## Methods

### Eligibility Criteria

The PRISMA-ScR (Preferred Reporting Items for Systematic Reviews and Meta-Analyses extension for Scoping Reviews) checklist was followed to ensure the reliability of our results [13]. To meet the eligibility criteria, the included papers had to (1) address the design or evaluation of digital health, (2) report increased well-being as the purpose of digital health, (3) address the patient as the main user, and (4) be published in English. Digital health is mainly designed to improve the well-being of patients. We particularly focused on patients as the main users to locate our research in the context of health care. Age or other criteria were not attributed to the type of patient considered. However, we will reflect on the other users, such as caregivers, who are considered in the design and evaluation processes, in addition to the patient. All types of original peer-reviewed research papers were considered. Reviews were excluded from the search as they did not provide in-depth insights into the 4 guiding problems for each digital health case. To obtain a complete overview of all digital health papers, we did not include any inclusion restrictions on the publication period (see Textbox 1 for an overview of the eligibility criteria).

**Eligibility criteria for the systematic scoping review.**

**Inclusion criteria**

- Study types: any type of original peer-reviewed research paper
- Period: any paper published before February 24, 2021
- Language: English
- Population: any patient receiving care for prevention, cure, or rehabilitation
- Intervention: the design or evaluation of digital health technology
- Outcome: the well-being of the patient

**Exclusion criteria**

- Study types: conference abstracts, (systematic) reviews, opinion papers, editorials, doctoral theses, workshops, protocols, and textbooks
- Period: papers published after February 24, 2021
- Language: all other languages
- Population: healthy people, health in the workplace, and health of caregivers
- Intervention: no interaction among patient and technology, medical aids (such as electronic wheelchair or intravenous pump), does not address design or evaluation of digital technology, or technical description of the technology
- Outcome: the digital technology does not aim to improve well-being; digital technology only aims to log well-being without considering the impact on well-being and well-being only referred to in the abstract and not in full text

### Search Strategy

Papers were collected from six databases on February 24, 2021: ACM Digital Library, PubMed, Web of Science, IEEE Xplore, PhilPapers, and Google Scholar. We used a combination of the following terms appearing in the title or abstract of the papers: (*wellbeing* OR *well-being*) AND (patient*) AND (*design* OR moral* OR ethic*) AND (*technology* OR *digital* OR *ehealth* OR *mhealth* OR *telemedicine* OR *telehealth* OR *electronic health* OR *mobile health* OR mobile* OR smart* OR *internet*; Multimedia Appendix 1). The terms *moral* and *ethic* were added to the search strategy as these terms might result in insight into various values related to well-being. The term *design* in this section was used as a synonym for the term *technology* in digital health.

### Study Selection

After collecting all papers from the databases, duplicates were removed. The titles and abstracts of the remaining papers were screened for eligibility criteria using Rayyan software [14] by 2 authors independently (MS and CK; Textbox 1). The full texts of the remaining papers were downloaded and independently screened for inclusion by the same authors. In both phases, the authors discussed disagreements until consensus was reached.

### Data Analysis

Data were extracted by 1 author (MS), who was guided by the 4 theoretical problems and their subquestions to understand if and how these problems are considered in design and evaluation practices (see Textbox 2 for subquestions for each problem directly derived from Brey [10]). The papers were divided into two groups based on their main focus: design and evaluation of digital health. Sections of each paper were marked when they were related to 1 of the 4 problems and their corresponding questions. All marked sections were grouped by question. Consequently, we compared the results of the various papers for each question to understand how each major problem was considered in practice. To determine what values constitute well-being in the epistemological problem, we followed the principles of thematic content analysis [15]. We coded each definition of well-being and categorized them into 7 overarching values. The results are presented for each problem.

**Categories and guiding questions for data extraction in the systematic scoping review.**

**General information**

- Type of research: design or evaluation
- Year
- Journal

**Epistemological problem**

- What values constitute well-being?
- Who defined well-being?
- What research tools are used to understand user well-being?

**Scope problem**

- Who is the main user of the technology?
- What other users are involved?
- What time span of well-being is considered?

**Specification problem**

- What type of technology is designed?
- What method is used to design or evaluate digital health?
- How is well-being translated into design requirements?

**Aggregation problem**

- Does the paper refer to conflicts in well-being?
- What type of conflicts are considered?
- How are conflicts solved?

## Results

### Study Selection

**Figure 1:**
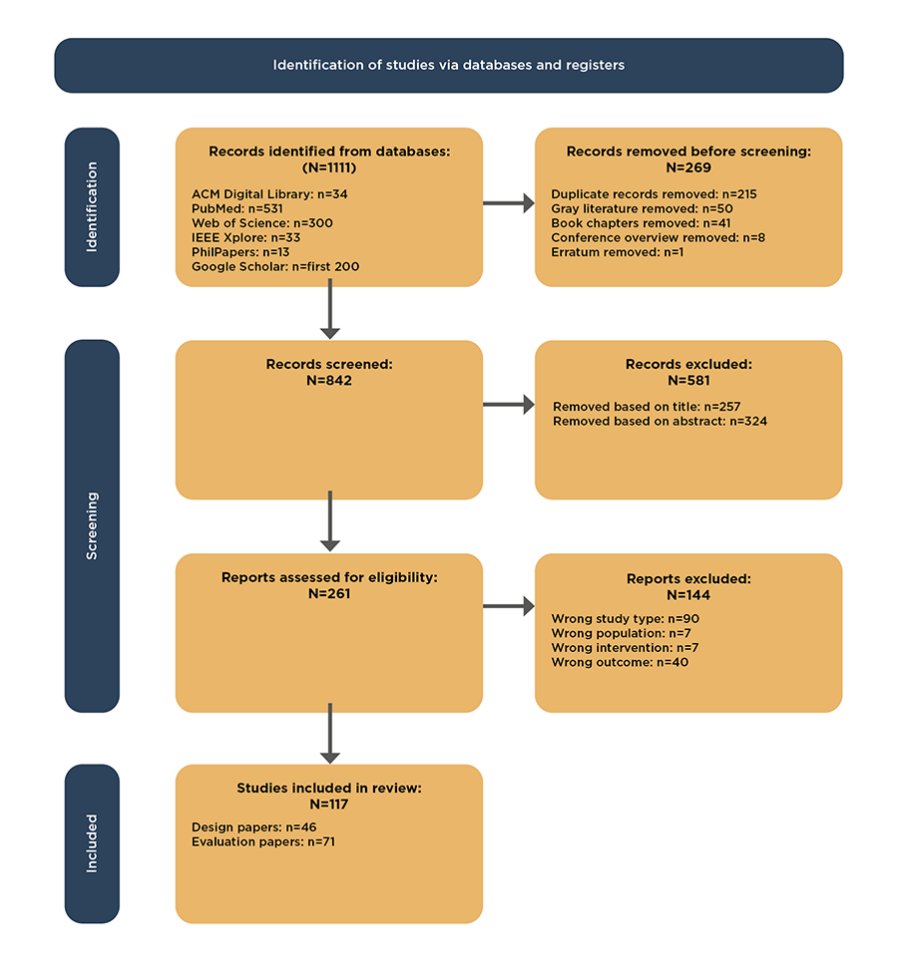
Flowchart of paper selection.

A total of 1111 papers were identified. After removing duplicates and gray literature, 75.79% (842/1111) of papers remained. After title and abstract screening, 69% (581/842) of papers were discarded mainly as they did not study a digital health intervention or did not consider the patient as the main user of the technology. Of the remaining 261 papers, full-text screening resulted in the exclusion of 90 (34.5%) papers for not being original research, 40 (15.3%) papers for only reporting well-being but not considering it in the design or evaluation process, and 14 (5.4%) papers for either not considering patients as the main user or digital health as an intervention (see Figure 1, which was derived from the PRISMA [Preferred Reporting Items for Systematic Reviews and Meta-Analyses] 2020 flow diagram [16]).

Of the 261 papers, 117 (44.8%) papers remained for data extraction, of which 46 (39.3%) papers focused on the design of digital health, and 71 (60.7%) addressed its evaluation. Although some papers described both processes, it was possible to group them according to their main focus. The included papers were published between 1999 and 2021. Approximately 80.3% (94/117) of all the papers were written in or after 2015.

### General Results

The results were presented for each of the 4 problems (see Table 1 and Table 2 for a summary of the general results and Multimedia Appendix 2 [17-133] for more detailed information).

**Table 1:** Overview of results for design papers^a^ (N=117).

Categories, review questions, and characteristics				Design papers (n=46), n (%)
**General information**				
	**Year**			
		After 2010	45 (98)	
		After 2018	24 (52)	
	**Journal**			
		JMIR or partner journals	10 (22)	
		Other journals	36 (78)	
**Epistemological problem**				
	**What is the definition of well-being?**			
		Healthy body	21 (46)	
		Healthy mind	15 (33)	
		Happy me	14 (30)	
		Social me	15 (33)	
		Self-managing me	21 (46)	
	**Who defined well-being?**			
		Author	31 (67)	
		User	15 (33)	
	**If users defined well-being, what research tools are used?**			
		Interview	11 (24)	
		Survey	2 (4)	
		Data derived from smartphone	1 (2)	
		Focus group and workshop	4 (9)	
		Observation	0 (0)	
**Scope problem**				
	**Who is the main user of the technology?**			
		Neoplasms	3 (7)	
		Mental, behavioral, and neurodevelopmental disorder	10 (22)	
		Endocrine, nutritional, or metabolic diseases	5 (11)	
		Other	28 (60)	
	**What other users are involved?**			
		No other users involved	11 (24)	
		Involvement of caregiver	26 (57)	
		Involvement of relatives	8 (17)	
		Involvement of experts	18 (39)	
	**What time span of well-being is considered?**			
		N/A^b^	N/A	
**Specification problem**				
	**What type of technology is designed?**			
		Support platform	30 (65)	
		Sensor	8 (17)	
		Phone or video support	0 (0)	
		Other	8 (18)	
	**What method is used to design or evaluate digital health?**			
		Interviews	16 (35)	
		Focus group	13 (28)	
		Usability test	15 (33)	
		Other	2 (4)	
	**How is well-being translated into design requirements?**			
		Not specified but generally based on insights from user research	—^c^	
**Aggregation problem**				
	**Does the paper refer to conflicts in well-being?**			
		Yes	11 (14)	
		No	35 (76)	
	**What type of conflicts are considered?**			
		Value conflicts within one user	1 (2)	
		Value conflicts within users of the same group	2 (4)	
		Value conflicts between users of different groups	10 (22)	
	**How are conflicts solved?**			
		Personalization	20 (43)	

^a^Some papers are categorized in multiple domains, which might result in percentages of >100%. This table only visualizes the most common outcomes. For more detailed information, see Multimedia Appendix 2 [17-133] and the *Results* section.

^b^N/A: not applicable.

^c^Not available.

**Table 2:** Overview of results for evaluation papers^a^ (N=117).

Categories, review questions, and characteristics						Evaluation papers (n=71), n (%)
**General information**						
	**Year (from 1999 to 2021)**					
		After 2010			67 (94)	
		After 2018			42 (59)	
	**Journal**					
		JMIR or partner journals			11 (15)	
		Other journals			60 (85)	
**Epistemological problem**						
	**What is the definition of well-being?**					
		Healthy body			28 (39)	
		Healthy mind			42 (59)	
		Happy me			35 (49)	
		Social me			28 (39)	
		Self-managing me			20 (28)	
		Functional me			12 (17)	
		External conditions			6 (8)	
	**Who defined well-being?**					
		**Author**			53 (75)	
			Without QoL^b^ questionnaire	5 (7)		
			With QoL questionnaire: a total of 154 different QoL questionnaires were used, of which 14 were disease specific)	48 (68)		
			PHQ-9^c^ used most often	8 (11)		
		User			22 (31)	
	**If users defined well-being, what research tools are used?**					
		Interview			9 (13)	
		Survey			10 (14)	
		Data derived from smartphone			1 (1)	
		Focus group and workshop			0 (0)	
		Observation			3 (4)	
**Scope problem**						
	**Who is the main user of the technology?**					
		Neoplasms			11 (15)	
		Mental, behavioral, and neurodevelopmental disorder			10 (14)	
		Endocrine, nutritional, or metabolic diseases			6 (8)	
		Other			44 (63)	
	**What other users are involved?**					
		No other users involved			59 (83)	
		Involvement of caregiver			9 (13)	
		Involvement of relatives			3 (4)	
		Involvement of experts			0 (0)	
	**What time span of well-being is considered?**					
		During use			13 (18)	
		Directly after use			43 (61)	
		0 to 1 month after use			4 (6)	
		1 to 3 months after use			11 (15)	
		3 to 6 months after use			10 (14)	
		6 to 12 months after use			9 (13)	
**Specification problem**						
	**What type of technology is designed?**					
		Support platform			38 (54)	
		Sensor			7 (10)	
		Phone or video support			12 (17)	
		Other			14 (19)	
	**What method is used to design or evaluate digital health?**					
		Pre-post measurement			23 (32)	
		Randomized controlled trial			22 (31)	
		Other			26 (37)	
	**How is well-being translated into design requirements?**					
		N/A^d^			N/A	
**Aggregation problem**						
	**Does the paper refer to conflicts in well-being?**					
		Yes			5 (7)	
		No			66 (93)	
	**What type of conflicts are considered?**					
		Value conflicts within one user			1 (1)	
		Value conflicts within users of the same group			4 (6)	
		Value conflicts between users of different groups			1 (1)	
	**How are conflicts solved?**					
		Personalization			31 (44)	

^a^Some papers are categorized in multiple domains, which might result in percentages of >100%. This table only visualizes the most common outcomes. For more detailed information, see Multimedia Appendix 2 [17-133] and the *Results* section.

^b^QoL: Quality of Life.

^c^PHQ-9: Patient Health Questionnaire-9.

^d^N/A: not applicable.

### Epistemological Problem

#### Overview

The epistemological problem refers to the definition of well-being in digital health, which defines well-being and the methods used to understand user well-being (Table 3).

**Table 3:** Epistemological problem of well-being in digital health (N=117).^a^

Values of well-being and who defined well-being			Design methodology (n=46)		Evaluation methodology (n=71)
**Healthy body**					
	Defined by author	[17-31]		—^b^	
	Defined by user	Interview [32-36] Workshop [37] Focus group [34]		Interview [38,39] Survey [40-42]	
	Validated questionnaire	—		Big Five Inventory [43] Checklist Individual Strength [44] EQ-5D^c^ [45] HRQoL^d^ [46] HRQoL-MacNew^e^ [47] LTPAQ^f^ [48] PAIS^g^ [49] Parkinson’s Disease Questionnaire-39 [50] Pittsburgh Sleep Quality Index [51] PWI-A^h^ [52] Quality of Well-being Scale [53] Multilevel Assessment Instrument [49] Short Form-12 Health Survey [45] Timed Up and Go Test [54] WHO-QOL BREF^i^ [55] 6-Minute Walking Test [54] QoLAD^j^ (Alzheimer disease) [56] Short Form-36 Health Survey [46,54,57,58] SwQoR^k^ [59] BCTRI^l^ (breast cancer) [49] FACT-B^m^ (breast cancer) [60,61] QoLBC^n^ (breast cancer) [62] Chronic Respiratory Disease Questionnaire (chronic respiratory disease) [63] Chronic Respiratory Questionnaire (chronic respiratory disease) [63] IVI-VLV^o^ (low vision) [64,65]	
**Functional me**					
	Defined by author	—		—	
	Defined by user	—		Survey [66]	
	Validated questionnaire	—		EQ-5D [45] Instrumental activities of daily living [54] MFHW^p^ (German) [67] MIDAS^q^ [68] HRQoL [46] HRQoL-MacNew [47] Parkinson’s Disease Questionnaire-39 [50] Sheehan Disability Scales [69] Short Form-12 Health Survey [45] Short Form-36 Health Survey [46,54,57,58] FACIT^r^ (chronic illness) [70]	
**Healthy mind**					
	Defined by author	[17,24,26,31,35,71-73]		—	
	Defined by user	Interview [34,74-76] Focus group [34] Survey [77,78] Data derived from smartphone [79]		Interview [80] Survey [40,41,81,82] Observation [83]	
	Validated questionnaire	—		Beck Depression Inventory [51,82] BRIEF-A^s^ [84] Depression, Anxiety, and Stress Scale-21 [52,85] EQ-5D [45] EuroQol-5 Dimension [86] Generalized Anxiety Disorder-7 [69,86,87] HADS^t^ [44,58,88-92] HRQoL [46] HRQoL-MacNew [47] Kessler 10-Item Scale [69] Paced Auditory Serial Addition Test [84] PCL-C^u^ [51] Parkinson’s Disease Questionnaire-39 [50] Patient Health Questionnaire-9 [48,69,84-87,93,94] Patient Health Questionnaire-15 [86] Perceived Stress Scale [51] Response to Stressful Experiences Scale [51] Short Form-12 Health Survey [45] Short Form-36 Health Survey [46,54,57,58] The Short Health Anxiety Inventory [86] Social Readjustment Rating Scale [82] Simon Task [84] SwQoR [59] Wechsler Adult Intelligence Scale-4 [84] WEMWBS^v^ [95,96] 5-Item Well-Being Index [97,98] WHO-QOL BREF [55] Work and Social Adjustment Scale [86] Zung Self‐Rating Anxiety Scale [99] Zung Self‐Rating Depression Scale [99] QoLAD (Alzheimer disease) [56] BCTRI (breast cancer) [49] QoLBC (breast cancer) [62] FACIT (chronic illness) [70] Chinese 15-item Diabetes Distress Scale (diabetes) [85] Diabetes Distress Scale (diabetes distress) [93] IVI-VLV (low vision) [64,65] SCI-QoL^w^ (spinal cord injury) [48,87]	
**Happy me**					
	Defined by author	[20,23,100-104]		—	
	Defined by user	Interview [32,74-76] Focus group [105] Workshop [37] Data derived from smartphone [79]		Interview [38,106,107] Survey [40,81,82,108-110] Observation [83,111]	
	Validated questionnaire	—		Acceptance and Action Questionnaire-2 [96] Client Satisfaction Questionnaire [92] Fordyce Happiness Scale [88] General Self-efficacy Scale [84] HeiQ^x^ [112] Herth Hope Scale [99] Humor Styles Questionnaire [43] MFHW (German) [67] Meaning in Life Questionnaire [99] Orientation to Happiness Scale [52] PAL-C^y^ [49] Positive and Negative Affect Schedule [43,52] PWI-A [52] Self-Compassion Scale [51] Subjective Happiness Scale [113] Self‐transcendence Scale [99] Satisfaction With Life Scale [52] Visual Analog Scale mood [88] WEMWBS [95,96] 5-Item Well-Being Index [97,98] WHO-QOL BREF [55] QoLAD (Alzheimer disease) [56] FACT-B (breast cancer) [60,61] QoLBC (breast cancer) [62] FACIT Spiritual Well-being scale–12 (chronic illness) [83] Diabetes Distress Scale (diabetes) [93] ICECAP-O^z^ (older adults) [45] IVI-VLV (low vision) [64,65]	
**Social me**					
	Defined by author	[20,25,71,100-102,114-119]		[120]	
	Defined by user	Interview [36,76] Focus group [105]		Interview [38,80,121] SMS text message analysis [122] Observation [121]	
	Validated questionnaire	—		Client Satisfaction Questionnaire [58] EQ-5D [85] HeiQ [112] HRQoL-MacNew [47] Multidimensional Scale of Perceived Social Support [51,110,113] PAIS [49] PAL-C [49] Parkinson’s Disease Questionnaire–39 [50] PWI-A [52] Dyadic Adjustment Scale [51] Short Form-12 Health Survey [45] Short Form-36 Health Survey [46,54,57,58] Ability to participate in Social Roles and Activities [48] University of California Los Angeles Loneliness Scale [110,113] WHO-QOL BREF [55] QoLAD (Alzheimer disease) [56] FACT-B (breast cancer) [60,61] QoLBC (breast cancer) [62] FACIT (chronic illness) [70] Diabetes Distress Scale (diabetes) [93] ICECAP-O (older adults) [45] SCI-QoL (spinal cord injury) [48,87]	
**Self-managing me**					
	Defined by author	[18-20,25,26,71,100,101,114-119,123,124]		[125-128]	
	Defined by user	Interview [34,76,129] Focus group [34,105,130]		Interview [38,54,80,106,107,121,131] Observation [121] Survey [81,132] Data derived from smartphone [133]	
	Validated questionnaire	—		20-item Diabetes Empowerment Scale [85] EQ-5D [45,85] HeiQ [112] SwQoR [59] QoLBC (breast cancer) [62] Diabetes Distress Scale (diabetes) [93] 14-item Summary for Diabetes Self-care Activities (diabetes) [85] ICECAP-O (older adults) [45]	
**External conditions**					
	Validated questionnaire	—		PAIS [49] PWI-A [52] WHO-QOL BREF [55] QoLAD (Alzheimer disease) [56] QoLBC (breast cancer) [62] ICECAP-O (older adults) [45]	

^a^Quality of life questionnaires were downloaded and analyzed by the authors and grouped according to the values of well-being that they considered.

^b^Not available (no papers found in this category).

^c^EQ-5D: EuroQol 5-Dimensions.

^d^HRQoL: Health-Related Quality of Life.

^e^HRQoL-MacNew: The MacNew Heart Disease Health-Related Quality of Life.

^f^LTPAQ: Leisure Time Physical Activity Questionnaire.

^g^PAIS: Psychosocial Adjustment to Illness Scale.

^h^PWI-A: Personal Well-being Index (Australian Version).

^i^WHO-QOL BREF: World Health Organization Quality of Life Questionnaire–Brief.

^j^QoLAD: Quality of Life in Alzheimer’s Disease.

^k^SwQoR: Swedish web version of the Quality of Recovery Questionnaire.

^l^BCTRI: Breast Cancer Treatment Response Inventory.

^m^FACT-B: Functional Assessment of Cancer Therapy–Breast.

^n^QoLBC: Quality of Life in Breast Cancer Patients.

^o^IVI-VLV: Vision Impairment–Very Low Vision Questionnaire.

^p^MFHW: Marburger Screening for Habitual Well-being (German).

^q^MIDAS: Migraine Disability Assessment Questionnaire.

^r^FACIT: Functional Assessment of Chronic Illness Therapy.

^s^BRIEF-A: Behavior Rating Inventory of Executive Function–Adult Version.

^t^HADS: Hospital Anxiety and Depression Questionnaire.

^u^PCL-C: Posttraumatic Stress Disorder Checklist–Civilian version.

^v^WEMWBS: Warwick–Edinburgh Mental Well-being Scale.

^w^SCI-QoL: Spinal Cord Injury Quality of Life subscales.

^x^HeiQ: Health Education Impact Questionnaire.

^y^PAL-C: Profile of Adaptation to Life Clinical Scale.

^z^ICECAP-O: ICEpop Capability measure for Older people.

#### Values Constituting Well-being

##### Overview

**Figure 2:**
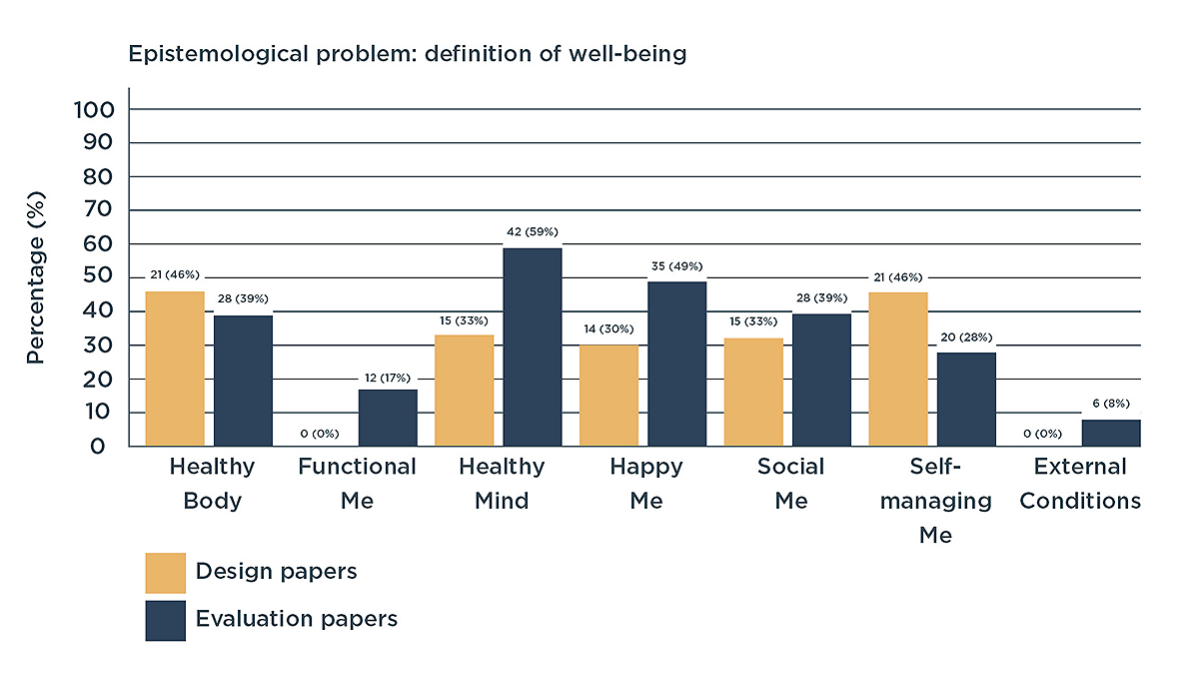
Values of well-being in design and evaluation papers (in number of papers and percentage of the total amount of papers in each process).

Papers that considered well-being in their study design were often vague in their definitions. For example, Kayrouz et al [69] studied digital health as part of the *well-being course* but did not define well-being. In such cases, we derived the definition from the research tools used in the papers. Most papers applied terms such as *mental well-being*, *emotional well-being*, or *spiritual well-being*. In addition, combinations of these words were used, as follows: *psychosocial well-being*, *psychospiritual well-being,* or *biopsychosocial well-being*. In comparing the different definitions of well-being, we found that similar terms did not always refer to the same content and that 2 varying terms were used to say the same thing. For example, spiritual well-being was used to refer to not only self-transcendence [99] but also self-acceptance [83]. In addition, depression and anxiety were termed interchangeably as belonging to the domains of *emotional well-being* [90], *mental well-being* [87], and *psychophysical well-being* [89]. For these reasons, we refrained from using these terms. Instead, we categorized the various definitions of well-being into values. In this review, we related values to what people consider important for obtaining well-being. After thematic content analysis, we identified seven values: *healthy body*, *functional me*, *healthy mind*, *happy me*, *social me*, *self-managing me*, and *external conditions* (Figure 2).

##### Healthy Body

This value of well-being was commonly considered in design and evaluation papers. It relates to health in its traditional definition: the absence of disease in the body. In the papers, this item was often referred to as *physical well-being*. Within this value, it is common to study the effect of disease on items such as pain experience, obesity, incontinence, sleep quality, vital sign monitoring, delirium, and sexual functioning.

##### Functional Me

*Functional me* refers to the ability to execute activities of daily living and reach life goals affected by one’s health condition. This value of well-being was identified based on a set of validated questionnaires used in evaluation papers, of which the 36-item Short Form Health Survey was used the most. None of the design papers considered *functional me* explicitly in their design.

##### Healthy Mind

A *healthy mind* was considered regularly in design papers and even more in evaluation papers. It referred to the absence of mental disease and was, in papers, mostly referred to as *mental well-being* or *emotional well-being*. Depression and anxiety are typical items that relate to this value. In addition, the effects of mental disease belong to this value, such as the effects of bipolar disorder, posttraumatic stress disorder, schizophrenia, and (mild) cognitive decline.

##### Happy Me

This value was considered by one-third of all design papers and half of all evaluation papers. It comprised the ability to feel happy, flourish, have a meaningful life, and accept one’s own body. In the reviewed papers, this value was often referred to as *psychological well-being*, *subjective well-being*, *emotional well-being*, *spiritual well-being*, *happiness*, and *wellness*. Items that are often considered related to this value are hope, satisfaction, positive experiences, pleasure, fulfilling personal potential, feeling needed, self-transcendence, and personal growth. Other important items are the ability to cope with and accept one’s own health status, self-confidence, being proud, feeling dignity, and not feeling stigmatized.

##### Social Me

Social Me was considered in approximately one-third of all design and evaluation papers. It includes all personal relationships that people have and the evaluation of these relationships. This value was consistently termed in the papers as *social well-being*. Items belonging to this value are related to conversations, feelings of partnership and friendship, compassion, trust, empathy, and support. Relationships studied within the papers included those with partners, family members, friends, and health care providers.

##### Self-managing Me

Approximately half of all design papers considered this item compared with fewer evaluation papers. *Self-managing me* relates to the ability to understand and manage one’s health care condition autonomously. Only *psychological well-being* was sometimes related to this value; no other specific terms were used within the papers. Concepts such as autonomy, competence, confidence, free will, decision-making, empowerment, and self-understanding belong to this category. The ability to understand and make use of health care information to make well-informed decisions, or *health literacy*, is also important in this context.

##### External Conditions

External conditions do not directly refer to a personal ability as other values do. In addition, this item was not studied in design and was rarely studied in evaluation papers. Nonetheless, we included it as several evaluation questionnaires dedicated questions to these conditions, and these could not be grouped elsewhere. Such questions referred to external conditions that created the setting for the well-being of patients and included, among others, financial security and having a job and a house.

#### User Definition of Well-being

In most design and evaluation papers, well-being was solely defined by the authors (eg, researchers, physicians, designers, and engineers). Design papers commonly referenced the literature to justify their definitions of well-being. Evaluation papers made use of validated quality of life (QoL) questionnaires, from which the authors derived a definition of well-being. A total of 154 unique questionnaires on well-being were found in the evaluation papers. Of these 154 questionnaires, 14 (9.1%) could only be applied to a specific disease. In only one-third of all design and evaluation papers, the main users of digital health were questioned about their understanding of well-being. In these cases, users were often not *free* to define the several values of well-being but were questioned on how they considered a specific item, such as *physical well-being*. Design papers considered these user definitions in their design requirements. Evaluation papers considered these as a qualitative outcome of the evaluation process. In general, these definitions of users do not seem to vary from the definitions provided by the authors.

#### Research Tools Used for Understanding User Well-being

In the few papers in which users provided insight into what they considered as well-being, several empirical research tools were used. The most common tools used to understand users’ conceptualization of well-being were interviews and open nonvalidated surveys. Design papers commonly considered interviews or workshops. Evaluation papers mostly considered surveys. In 1.7% (2/117) of papers, the concept of well-being was defined through data collected based on smartphone use [79,122].

### Scope Problem

To understand how the scope problem is considered in the design and evaluation of digital health, we studied the main users of the technology, the additional users, and the time frame considered.

#### Main User of the Technology

**Figure 3:**
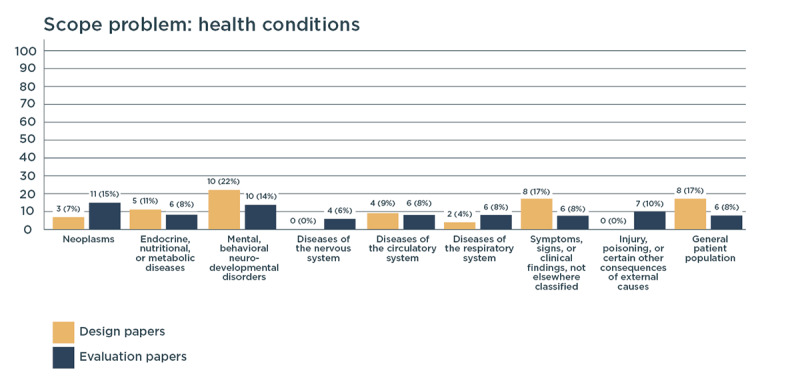
Disease classification of patients in design and evaluation papers (in number of papers and percentage of the total amount of papers in each process).

We grouped the health conditions of the main users of digital health (eg, patients) according to the International Classification of Diseases for Mortality and Morbidity [134] (Figure 3). Design papers showed that most digital health solutions were created for patients with mental disorders, in particular depression, anxiety, and cognitive decline. Evaluation papers mostly considered mental disorders and neoplasms.

#### Other Users Involved

**Figure 4:**
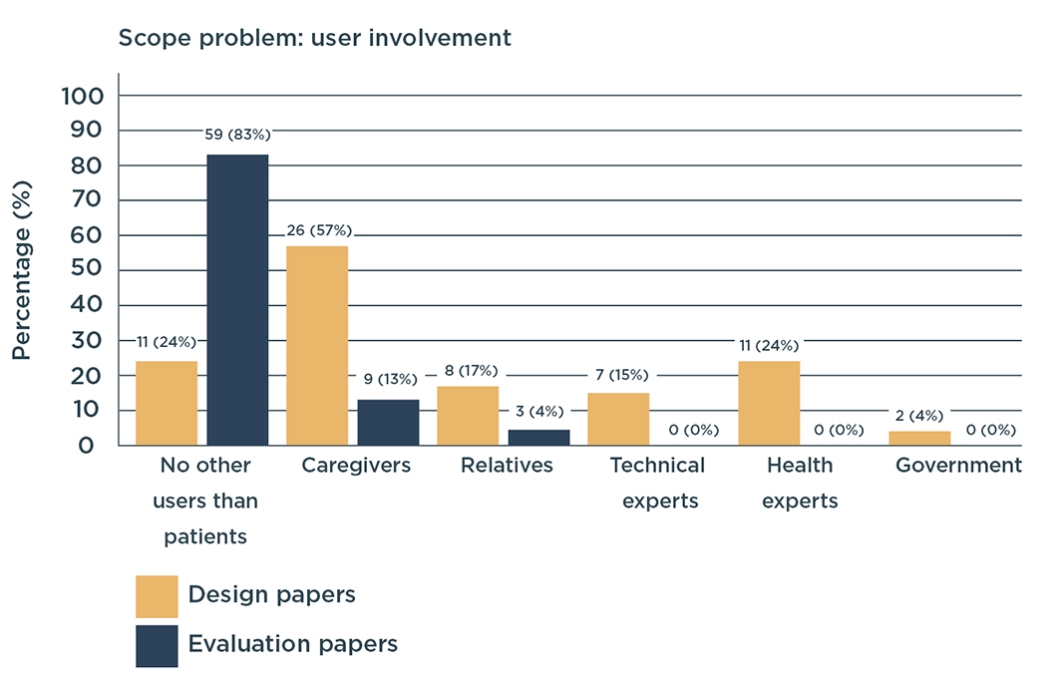
Other users considered in design and evaluation papers (in number of papers and percentage of the total amount of papers in each process). Categories with fewer than 4 papers in total were left out of the figure.

In addition to the main users—patients—we studied what other users were considered in each paper. Design papers commonly considered other users (eg, caregivers and relatives). In contrast, only a few evaluation papers considered other users. The reason for involving other users in the papers was mainly to provide input regarding patients’ needs rather than their own. Recognizing the value of involving a wide variety of users in design, 4% (2/46) of design papers conducted an in-depth exploration of the types of users to involve in the design process [35,118] (Figure 4).

#### Time Span of Well-being

In evaluation papers, the effect of technology on well-being was mostly measured during the use of the technology or on the day directly after its use. Only a few papers conducted an additional measurement after 1 to 12 months from initial use.

### Specification Problem

The specification problem relates to the embodiment of well-being in the design of digital health. To achieve this, we extracted the type of technology, the design or evaluation method used, and the procedure for creating design requirements*.*

#### Type of Technology

**Figure 5:**
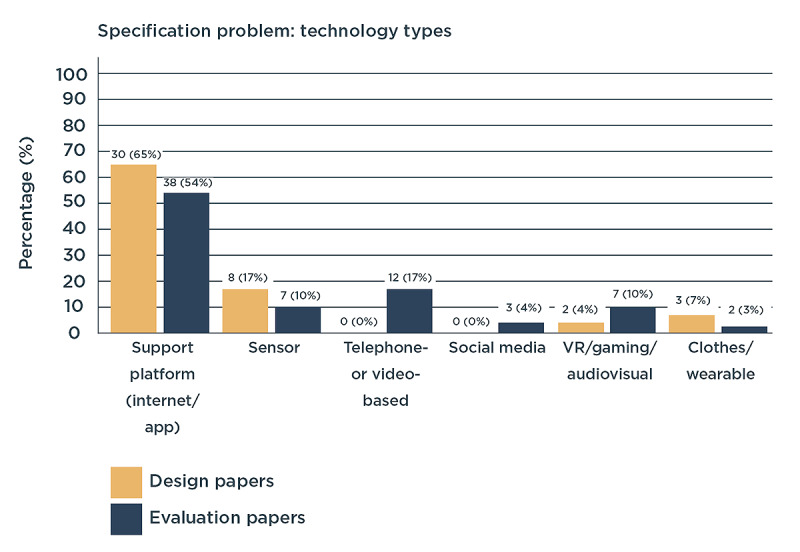
Technology types considered in design and evaluation papers (in number of papers and percentage of the total amount of papers in each process). VR: virtual reality.

Most design and evaluation papers studied the application of a supporting digital platform. Such a platform is provided to users through the internet or via a tablet or smartphone app. Few design papers also considered the design of sensors or wearables. Other technologies considered by the evaluation papers were mainly telephone- or video-based consulting and support (Figure 5). The great majority of the digital interventions were designed and evaluated for use at home. Only very few were found to be used within the hospitals, primary care settings, or public spaces.

#### Method for Designing and Evaluating Digital Health

In design papers, a wide range of methodologies was used to create digital health, and most of them were focused on the inclusion of the user in the design process through participatory design. Interviews, focus groups, and workshops were regularly used. In addition, usability testing through small pilot studies and prototype testing were popular tools for the design and refinement of technologies. Methods structuring the design process greatly varied and included, for example, the methods of service design for value networks [118], persona enrichment process [119], social network analysis [35], systems development [19], transformative service research [100], and human factors research [20,101]. In addition, numerous papers applied varying frameworks to design for behavior change [18,22,25,26,32,34,116].

In comparison with design papers, evaluation papers were more consistent in their methodologies. Most papers applied a pre–post study design or a randomized controlled trial (RCT) to evaluate the digital health intervention compared with a baseline or control group. Another type of evaluation paper considered interviews to understand the usability and acceptability of the technology, sometimes in addition to a pre–post study or an RCT. More rare forms of evaluation were population surveys [81,127,128] or analysis of technology use through big data analysis [33,48,91,122]. An alternative to the RCT was explored once, named *partially randomized patient preference*. In partially randomized patient preference, patients were allocated based on their preference in either the intervention or control group. The authors concluded that the intervention has higher efficacy when patients have consciously chosen for its use [58].

#### Translation Into Design Requirements

Design papers commonly did not explain the methodology used to translate user input into design requirements. We only identified a few papers that illustrated their procedures. For example, requirements were created by coding user input into requirements [101], and the requirements were explicitly elucidated by users in a workshop [37].

### Aggregation Problem

The aggregation problem refers to conflicts within the values of well-being. We aimed to understand if such value conflicts were reported and, if so, the type of conflicts that arose and the solution for solving these conflicts.

#### Conflicts in Well-being

**Figure 6:**
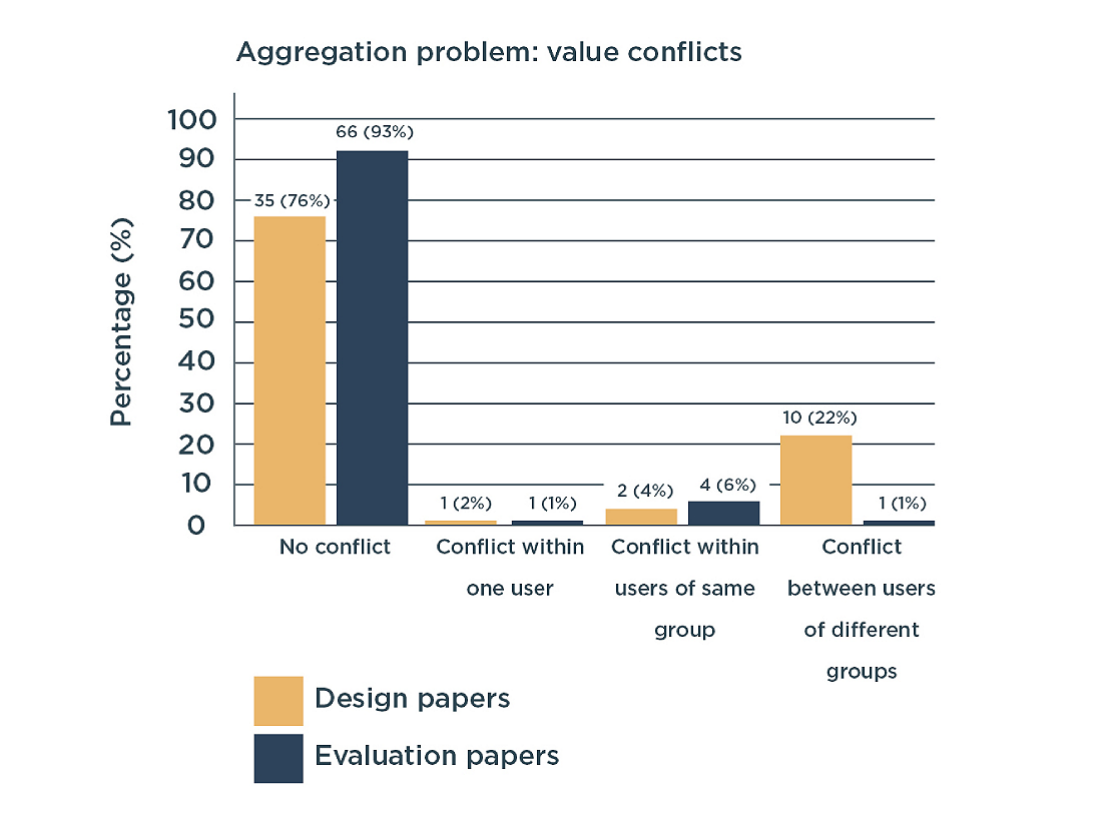
Value conflicts considered in design and evaluation papers (in number of papers and percentage of the total amount of papers in each process).

The great majority of papers did not consider value conflicts. Design papers considered conflicts more often than the evaluation papers (Figure 6). For example, Doherty et al [74] considered a set of value tensions as a source of design inspiration.

#### Type of Conflicts

Value conflicts within one user were explicitly considered in 1.7% (2/117) of papers. These papers studied both the benefits and harms of digital health for individual well-being [74,82]. For example, the ability to obtain support through technology versus the reduction in personal contact was contrasted. Approximately 4% (2/46) of design papers [74,130] and 6% (4/71) of evaluation papers [38,81,89,133] considered a conflict arising among users of the same group. Such conflicts generally referred to the differences among individual patients in their desire to apply digital health [38,89,133] or their ability to use it [81,130]. Conflicts among users of different groups were considered by 22% (10/46) of design papers and only 1% (1/71) of evaluation papers [127]. This difference might have resulted from the evaluation papers involving only patients and no other users in the study design. Such conflicts mostly occurred between patients and caregivers. For example, Kujala et al [127] illustrated the use of digital health to improve patient autonomy and decrease caregiver autonomy. Cahill et al [101] showed conflicts among organizational needs (ie, staff costs and keeping residents safe) and patient needs (ie, independence, privacy, and social interaction). In another paper, the same authors explained that although digital health might benefit patients, it might hinder nurses’ working processes [20]. Derboven et al [117] addressed a conflict in the autonomy of patients and control over patients by caregivers (Figure 6).

#### Conflict Resolution

Most studies only referred to conflicts without providing solutions. The few solutions offered were procedural, such as engaging in multidisciplinary collaborations [27], weighing the benefits and harms of conflicting values [117], and being aware of conflicts [102]. Another solution for solving value conflicts within users was offered by Doherty et al [75] and was related to personalizing digital health based on individual needs. The topic of digital health personalization was found in approximately half of all design and evaluation papers but was rarely explicitly related to the topic of value conflicts. Multiple personalization options were addressed. For example, papers recommended providing individualized health advice through digital health [17,23,57,66,77,78,122], adjusting software to patient needs [20,39,41,48,95,98,107,108,110,112,114,122,133], the importance of also supporting nonusers [18,38,44,75,81,89,91,113], and personalizing the support needed to apply digital health [35,49,56,59,100,101,117]. Other personalization options included changing motivational gaming techniques to individual needs [22,41,132], adapting solutions to specific cultures [60,129,133], allowing patients to make their own motivational messages [86], choosing the gender of the digital health assistant [124], and inserting personal memories into the design [99,111].

## Discussion

### Principal Findings

The enormous growth in papers since 2010 reflects that digital health is increasingly being applied to improve the well-being of patients. In addition, this type of technology is rapidly advancing. Although studies on digital health started with CDs and telephone consulting, today’s studies increasingly cover health care provision via web-based supporting platforms, social media, and remote sensing. In all of these cases, we identified how practice deals with the commonly discussed theoretical problems in technology for well-being. We defined common values related to well-being and identified that well-being was rarely defined by the users themselves. In addition, we identified that the current scope of well-being is generally small, involving few users in a short time frame. We illustrated that many methodologies exist on how to embed well-being in design, whereas only a few methods are accepted for evaluation. Finally, we identified that value conflicts, commonly discussed in theory [10,135], are rarely considered in practice. Our results show that simple solutions exist for solving theoretical problems that are paramount in technology for well-being. At the same time, this theory can challenge current practices for continuous improvement.

### Definition of Well-being

Within health care, health is considered a state of complete physical, mental, and social well-being. After the introduction of this definition in 1948, it was scarcely applied in practice [136,137] and regularly criticized for being unrealistic. Such criticisms mostly applied to the word *complete*, as this word implies that people cannot feel healthy without absolute physical, mental, and social well-being. In an aging society, *complete* well-being would then only be reserved for a few individuals [138]. To specify the novel definition of health as well-being, the concept of *positive health* was introduced by various authors [139-142]. Positive health focuses on health through well-being. It is the ability to *flourish* despite mental or physical diseases. In addition, as a response to assess health from the perspective of well-being, an entire range of validated questionnaires emerged, often termed QoL measurement scales [137]. Although used often in recent times, the terms of *well-being*, *positive health*, and *QoL* are *rarely defined*, which made Locker and Gibson [143] conclude that the commitment to these concepts is *more rhetorical than real*.

Our review sheds light on how well-being is conceptualized in the context of digital health provision. First, we found that, unfortunately, a large majority of papers only reported on well-being without actually implementing it, which corresponds to the rhetorical commitment to well-being postulated by Locker and Gibson [143]. In the design and evaluation papers that committed to well-being, we were able to identify seven values commonly considered as part of well-being: *healthy body*, *functional me*, *healthy mind*, *happy me*, *social me*, *self-managing me*, and *external conditions*. The often-occurring values of *healthy body*, *healthy mind*, and *social me* reflect the definition of health as physical, mental, and social well-being, as proposed by the World Health Organization. The value of *happy me* was found to greatly reflect the movement of positive health, aiming for health care to make a move from disease toward happiness and flourishing. Remarkably, we also commonly found *self-managing* me to be an important value of well-being related to digital health. Previously, this value was often not considered to be a part of patients’ well-being. Digital technologies have the ability to locate the center of health from hospital to home and from health care provider to patient. With this ability, they can increase the autonomy of patients. We believe that with the growing use of digital health, the importance given to the value of *self-managing me* will increase. As a consequence, it might be important to constantly reflect on the interactions among patients, technologies, and values of well-being to optimally design health care for patients’ well-being. The identified values can be used as a source of inspiration for digital health designers and evaluators to co-design technologies for optimal well-being with patients.

### Differences Between Design and Evaluation

We identified more evaluation papers than design papers. This difference might result from design research not always reporting on its processes [144]. In addition, we found differences between the design and evaluation papers regarding the 4 problems. Design papers mostly considered well-being as *healthy body* and *self-managing me*. Evaluation papers often considered the values of *healthy mind* and *happy me*. Design papers mainly focused on mental disorders. Evaluation papers also studied mental disorders and neoplasms. Other users, such as caregivers and relatives, were regularly involved in design papers but not in evaluation papers. Design papers used numerous design methods, whereas evaluation papers mostly considered pre-post measurements or RCTs as a method. Value conflicts were rarely described in design papers and even less in evaluation papers.

Clearly, differences exist between the processes of design and evaluation, given that they take place in different stages of the digital health development process. Design processes commonly take place outside the care context. These processes are both creative and exploratory. In contrast, evaluation follows the rules of *evidence-based health* and is located in the health care environment [145]. Evaluation is more bound to standardization and reflection. The different characteristics of both processes result in the need for different methodologies for design and evaluation, as discussed in the specification problem. However, the differences seem to be larger than what can be explained by the stage of development. One would expect that the design and evaluation of digital health consider an equal approach to epistemological and scope problems by adopting the same definition of well-being, the same ways of obtaining this definition, and focusing on the same user groups. Nevertheless, this review shows different results. Design and evaluation vary greatly in their definitions of well-being and the main patient groups that are commonly designed for. This is troublesome, considering, for example, an application for postoperative patients to monitor their health. The design team creates this solution to improve the value of *self-managing me* for these patients. During the evaluation, this technology is assessed based on its ability to improve the *healthy body* and the *healthy mind*. The misalignment between the design input and evaluation outcomes results in suboptimal insight into the potential of the technology, which could hinder its successful implementation. Currently, only a minority of digital health technologies have achieved successful implementation. More digital health technologies would achieve successful implementation when the design is better aligned with evaluation and vice versa [146,147]. In particular, we argue that design and evaluation should consider similar definitions of well-being in the design and evaluation processes and the same user groups.

### Methods of Creating Well-being

In most design and evaluation papers, well-being was defined without user input. When users were questioned, they were commonly asked to define only a specific value of well-being instead of explaining the values they considered important. Brey [10] previously noted that well-being could not be objectively determined independently from the user. Our results are worrisome, as the papers did not obtain a real understanding of what users considered to be important values belonging to their well-being. Involving users in defining the values of well-being as a source of design inspiration and evaluation outcome is recommended.

Methods for designing and evaluating digital health clearly differ. In most design papers, a participatory design was applied. However, as Orlowski and Matthews [148] argued, in addition to participatory design, a design method for structuring the process is needed. The papers in this review considered a wide variety of design methods to structure the design process. A review of the methods for usability testing of eHealth showed similar results [144]. No consistency seems to exist in the design papers on what design methods to use to design for well-being. The question of what design method to use to create well-being has been raised in the past [149]. Multiple papers have been written on the potential to embed the values of well-being in technology design. Design methods such as value-sensitive design [150] or values that matter [151] have been introduced. In addition, numerous papers have aimed at creating *design for well-being* methodologies to provide designers with a framework for embodying well-being in design. Examples in the literature are the approaches of emotional design [152]; life-based design [153]; capability-sensitive design [154]; positive design [155]; motivation, engagement, and thriving in user experience model [156]; positive technology [157]; experience design [158]; and positive computing [159]. Given this myriad of design methodologies that particularly aim at our goal of designing for well-being, it is remarkable that none of these methods was found to be used in the design papers. We speculate that this resulted from not knowing about the design methods, finding the methods difficult to apply to health care, or valuing well-known methods over novel ones. Future digital health designers would benefit from heuristics on which method to use in what situation. This can be facilitated by increasing awareness of design methods and transparency and reporting on the reasons for using a certain design method.

The interaction between users and technology and the context of use might affect how well-being is expressed, which does not necessarily correspond to the initial embodiment of well-being in design. This is called the *positivist problem* [160]. For that reason, an evaluation process is necessary to study the actual effects of technology on well-being. Evaluation papers commonly considered the same set of evaluation methodologies: pre-post measurements and RCTs.

The RCT methodology, which has become the gold standard for effectiveness studies in pharmacological interventions, has been transferred one to one to evaluate the effectiveness of nonpharmacological interventions, including digital health [147]. An RCT only provides valuable information on an intervention’s effectiveness when (1) the intervention and the way of providing the intervention are stable, (2) the intervention can be applied with fidelity, and (3) when it is expected that the outcomes of the intervention are measurable and meaningful [161]. Given these requisites, the use of RCTs in digital health has been subject to several concerns. An RCT requires that the intervention is stable, implying that the digital health technology has been finalized before the start of the RCT. However, design is an iterative process that requires numerous phases of testing and adaptation. Clinical outcomes obtained through an RCT are often required to justify the use of more resources to improve the design. However, when the design is improved based on the clinical outcomes of RCTs, these outcomes become directly outdated. Similarly, when a digital health solution needs to await clinical outcome measures for improvement, the technology might be outdated once the trial is finished [162]. In addition, a digital health solution cannot be directly applied with high fidelity as the solution mediates clinical processes and might require changes to the context of use to be optimally used. However, these effects require further study. The limited study design of an RCT does not permit the study of such mediating effects, and without knowing the optimal context of use, digital health cannot be applied with high fidelity [163].

Several changes and alternatives to RCTs have been proposed to align scientific evaluations with digital health. These include, among others, a multiphase optimization strategy for the RCT, allowing the design to be adapted during the evaluation process [164], evaluating the principle of a solution rather than the specific technology itself [162], and broadening the set of outcome measures into the inclusion of human–technology interactions [165] and legal and ethical evaluations [166,167]. Another interesting alternative is a single case experimental design [168]. This method illustrates that RCTs only provide an *average* good and not the optimal solution for each individual. They propose to observe a single case over a longer period while manipulating treatment (technology). This study design allows personalizing digital health to an individual’s needs, thereby increasing its efficacy. So far, the papers in this review have barely considered moving beyond traditional evaluation methods. Our results resemble previous results from systematic reviews of evaluation methods and health technology assessments of digital health [169,170]. There is a need for the use of evaluation methods that are better aligned with the complexity of digital health.

In addition, not only RCTs but also the many validated QoL questionnaires to assess the effect of digital health via an RCT need reflection. In the past, these questionnaires were studied thoroughly [137,171]. Guyatt et al [171] rightfully posed the concern of how to select, use, and interpret such questionnaires. We share their concerns and believe that when applying such a questionnaire, it is important to understand and illustrate what values of well-being are measured through the questionnaires and to be aware of the potential and limitations of what is being asked. Similarly, as Blandford et al [147] argued, such questionnaires only have a limited face and construct validity. They do not provide insight into how the social structures of care change and how technology is changing the current values of well-being [172]. In addition, QoL questionnaires were validated for a specific user group in a specific context. When technology is introduced, the entire basis on which such questionnaires are validated might change. Thus, the use of QoL questionnaires requires careful consideration of their selection and application. Our 7 values provide a comprehensive and up-to-date view of well-being. Further work could be done to develop tools (eg, questionnaires) based on the 7 values to complement and support the current evaluation methods to better align with the complexity of digital health.

### Recommendations for Best Practice

On the basis of the theoretical and practical reflections of this review, we sketched an *ideal practice* for anyone involved in the design and evaluation of digital health for well-being. This ideal practice begins with the formation of multidisciplinary teams working together from the start of the design process to successful implementation. After the composition of the team, we advise demarcating the scope of the project. Which users will be affected by the technology? What users will be studied, and who will be left out of the study’s scope? Furthermore, we encourage the upfront identification of the moments in time during which the effect of technology is evaluated. The process should continue with a clear study on the definition of well-being per user group in which users are closely involved. To prevent well-being from becoming a buzzword, the team should consider constantly aligning the following design and evaluation processes to the found values of well-being. Instead of two linear processes, design and evaluation take place simultaneously. Evaluation outcomes are the source of design input and vice versa. As many methods exist for designing for well-being, the team should decide together which method is most suitable. The reasoning process is reported to facilitate other teams to make their own decisions. To justify the complexity of digital health, the type of evaluation method is carefully considered. The chosen method enables the evaluation of the user–technology–value interaction in an authentic context of use. In addition, the method facilitates obtaining insight into individual experiences for the personalization of the solution.

### Strengths and Limitations

In this paper, we aimed to bridge the gaps between the practices of design and evaluation and the theory produced on digital health technologies aimed at improving well-being. This endeavor is both a strength and a limitation. By highlighting the differences among the fields, we enable the design to consider the context of evaluation and vice versa, inspire practice to consider better theoretical insights, and guide theorists to acknowledge the need for pragmatic decision-making in practice. The application of our recommendations would result in individual, social, and economic benefits. First, at the individual level, digital health would foster a culture of *inclusiveness* through personalization. It would better meet the needs and values of individual patients, caregivers, and relatives, regardless of their education, age, gender, or culture. Second, on a social level, digital health would fit within current health care practices and align with the care processes of health care personnel. Third, economic benefits will arise from the alignment of design with evaluation by preventing a waste of resources and leading to a more successful uptake of digital health.

A limitation of our study is that it was difficult to report insights from all disciplines using a common language. The attempt to place all insights from practice into theoretical frameworks might have resulted in missing important items or mistakenly interpreting certain practical phenomena as belonging to certain theoretical concepts. For example, we found that a great majority of design papers aimed to create digital health technologies for lifestyle management of the older population, regardless of their medical condition. By categorizing all design papers into the International Classification of Diseases for Mortality and Morbidity framework, these *older adults* were divided into three different categories (mental disorder, disease of the nervous system, and symptoms not classified elsewhere). Although design papers often considered lifestyle management and older adults, this insight was invisible to those looking at the graph only.

A second limitation is that we aimed to include a wide variety of digital health technologies, although our results mostly identified digital health solutions in the domain of support platforms via apps or the internet. In addition, almost all solutions were designed for use at home. This is remarkable, as our search strategy did not focus solely on the home. Obviously, the terms *telemedicine* and *telehealth* refer to technologies for use at home. Nonetheless, we also included the term *digital* in our search strategy, which we expected would result in papers on digital technologies used in hospitals and primary care settings. Our search strategy had missing terms that would result in papers on technology use in hospitals, technology use in hospitals is not yet common, or it is not discussed in relation to well-being. Although we believe that our insights can also be applied to a wider range of digital health technologies in varying contexts, in the future, it is interesting to expand our research to varying technologies and extend it to the inpatient care context to understand the differences in designing technologies in diverging contexts. Furthermore, as we only included papers focused on well-being improvement, we might have missed the results on in-person value conflicts that are reported in papers studying the harmful effects of digital health on well-being. Finally, we rarely found sets of papers that focused on the design and evaluation of the same technology. The ability to compare the design and evaluation processes of similar technologies would have led to more reliable results. In the future, better aligning design and evaluation would ideally also result in more consistent reporting.

### Conclusions

In this review, we have shown how current practices deal with the major problems that are paramount in digital health for well-being. We identified major gaps in design and evaluation regarding their conceptualization of well-being, types of users studied, and methods used to design and assess well-being. The comparison of empirical practice with theoretical frameworks also showed how both fields have found pragmatic ways of circumventing or dealing with the problems of digital health for well-being. By illuminating the differences between design and evaluation, as well as practice and theory, and providing recommendations for best practice, we expect to have set the first steps to slightly bridge some gaps. As digital health technologies are gaining an increasingly important role in the future, we believe that multidisciplinary collaborations are required to be improved by moving beyond the common dichotomy of design and evaluation. Only then it is possible to transcend from digital health toward digital well-being.

## Appendix

Multimedia Appendix 1 Search strategy.

Multimedia Appendix 2 Detailed results.

## Abbreviations

PRISMA: Preferred Reporting Items for Systematic Reviews and Meta-Analyses
PRISMA-ScR: Preferred Reporting Items for Systematic Reviews and Meta-Analyses extension for Scoping Reviews
QoL: Quality of Life
RCT: Randomized Controlled Trial
