# Supplementary material for: From Digital Health to Digital Well-being: Systematic Scoping Review
Source: J Med Internet Res. 2022 Apr 4;24(4):e33787. doi: 10.2196/33787 (PMC9016508; doi:10.2196/33787)
Supplement: Multimedia Appendix 1 [file jmir_v24i4e33787_app1.doc]

### Multimedia Appendix 1

Run on 24 of February 2021

| **Database** | **Where** | **Search command** | **Papers** |
| --- | --- | --- | --- |
| ACM digital library  [LINK](https://dl.acm.org/action/doSearch?fillQuickSearch=false&expand=dl&AllField=Abstract%3A((("well+being"+OR+wellbeing)++AND++(patient*)+AND++(design+OR+ethic*+OR+moral*)++AND++("technology"+OR+"ehealth"+OR+"mhealth"+OR+"telemedicine"+OR+"telehealth"+OR+"digital"+OR+"electronic+health"+OR+"mobile+health"+OR+"Mobile*"+OR+"smart*"+OR+"internet")))) | Abstract | ("well being" OR wellbeing)  AND  (patient*)  AND  (design OR ethic* OR moral*)  AND  ("technology" OR "ehealth" OR "mhealth" OR "telemedicine" OR "telehealth" OR "digital" OR "electronic health" OR "mobile health" OR "Mobile*" OR "smart*" OR "internet") | 34 |
| Pubmed  [LINK](https://pubmed.ncbi.nlm.nih.gov/?term=("wellbeing"%5BTitle%2FAbstract%5D+OR+"well+being"%5BTitle%2FAbstract%5D)+AND+("patients"%5BMeSH+Terms%5D+OR+"patient*"%5BTitle%2FAbstract%5D)+AND+("design*"%5BTitle%2FAbstract%5D+OR+"ethic*"%5BTitle%2FAbstract%5D+OR+"moral*"%5BTitle%2FAbstract%5D)+%0AAND+("technology"%5BMeSH+Terms%5D+OR+"technology"%5BTitle%2FAbstract%5D+OR+"ehealth"+%5BTitle%2FAbstract%5D+OR+"mhealth"+%5BTitle%2FAbstract%5D+OR+"telemedicine"%5BMeSH+Terms%5D+OR+"telemedicine"%5BTitle%2FAbstract%5D+OR+"telehealth"+%5BTitle%2FAbstract%5D+OR+"digital"+%5BTitle%2FAbstract%5D+OR+"electronic+health"+%5BTitle%2FAbstract%5D+OR+"mobile+health"+%5BTitle%2FAbstract%5D+OR+"mobile*"+%5BTitle%2FAbstract%5D+OR+"smart*"+%5BTitle%2FAbstract%5D+OR+"internet"+%5BTitle%2FAbstract%5D)&ac=no&sort=relevance) | Title/  Abstract/ mesh | **("wellbeing"[Title/Abstract] OR "well being"[Title/Abstract]) AND**  **("patients"[MeSH Terms] OR "patient*"[Title/Abstract])**  **AND**  **("design*"[Title/Abstract] OR "ethic*"[Title/Abstract] OR "moral*"[Title/Abstract])**  **AND**  **("technology"[MeSH Terms] OR "technology"[Title/Abstract] OR "ehealth" [Title/Abstract] OR "mhealth" [Title/Abstract] OR "telemedicine"[MeSH Terms] OR "telemedicine"[Title/Abstract] OR "telehealth" [Title/Abstract] OR "digital" [Title/Abstract] OR "electronic health" [Title/Abstract] OR "mobile health" [Title/Abstract] OR "mobile*" [Title/Abstract] OR "smart*" [Title/Abstract] OR "internet" [Title/Abstract])** | 531 |
| Web of Science  [LINK](https://apps.webofknowledge.com/summary.do?product=WOS&doc=1&qid=1&SID=C3iR9guJfZnodgFMZ5W&search_mode=AdvancedSearch&update_back2search_link_param=yes) | TS (topic) | TS =  (("Wellbeing" OR "Wellbeing")  AND  ("patient")  AND  ("design" OR "moral*" OR "ethic*")  AND  ("technology" OR "ehealth" OR "mhealth" OR "telemedicine" OR "telehealth" OR "digital" OR "electronic health" OR "mobile health" OR "Mobile*" OR "smart*" OR "internet")) | 300 |
| IEEEXplore  [LINK](https://ieeexplore.ieee.org/search/searchresult.jsp?action=search&newsearch=true&matchBoolean=true&queryText=("Abstract":“Well-being OR "Abstract":Wellbeing”) AND ("Abstract":“patient*”  ) AND ("Abstract":“design OR "Abstract":moral* OR "Abstract":ethic*”) AND ("Abstract":“technology” OR "Abstract":“ehealth” OR "Abstract":“mhealth” OR "Abstract":“telemedicine” OR "Abstract":“telehealth” OR "Abstract":“digital” OR "Abstract":"electronic health" OR "Abstract":"mobile health" OR "Abstract":“Mobile*” OR "Abstract":“smart*” OR "Abstract":“internet”)) | Abstract | “Wellbeing OR Wellbeing”  AND  “patient*”  AND  “design OR moral* OR ethic*”  AND  “technology” OR “ehealth” OR “mhealth” OR “telemedicine” OR “telehealth” OR “digital” OR "electronic health" OR "mobile health" OR “Mobile*” OR “smart*” OR “internet” | 33 |
| Philpapers  [LINK](https://philpapers.org/search/advanced.pl) | Not applicable | Patient AND wellbeing AND technology | 13 |
| Google Scholar  [LINK](https://scholar.google.com/scholar?hl=nl&as_sdt=1%2C5&as_vis=1&q=(("Well-being"+OR+"Wellbeing")+AND+(patient)+AND+("design"+OR+moral+OR+ethic)+AND+("technology"+OR+"ehealth"+OR+"mhealth"+OR+"telemedicine"+OR+"telehealth"+OR+"digital"+OR+"electronic+health"+OR+"mobile"+OR+"smart"+OR+"internet"))&btnG=) | Not applicable | (("Wellbeing" OR "Wellbeing") AND (patient) AND ("design" OR moral OR ethic) AND ("technology" OR "ehealth" OR "mhealth" OR "telemedicine" OR "telehealth" OR "digital" OR "electronic health" OR "mobile" OR "smart" OR "internet")) | 253.000 **first 200** |
|  |  | **TOTAL** | 1111 |
