# Supplementary material for: From Digital Health to Digital Well-being: Systematic Scoping Review
Source: J Med Internet Res. 2022 Apr 4;24(4):e33787. doi: 10.2196/33787 (PMC9016508; doi:10.2196/33787)
Supplement: Multimedia Appendix 2 [file jmir_v24i4e33787_app2.doc]

### Multimedia Appendix 2

**Scope problem**

*Table 1: Overview of the scope problem. Design papers are contrasted to evaluation papers. The table shows what health conditions were designed for. Health conditions are classified according to the International Classification of Diseases for Mortality and Morbidity (ICD-11) [134].*

|  | **Health condition** | **Design papers** n=46 | **Frequency**  n (%) | **Evaluation papers** n=71 | **Frequency**  n (%) |
| --- | --- | --- | --- | --- | --- |
|  |  |  |  |  |  |
| 1 | Certain infectious or parasitic diseases |  |  |  |  |
| 2 | Neoplasms | [26, 104, 119] | 3 (7%) | [43, 44, 46, 49, 54, 60-62, 70, 88, 99] | 11 (15%) |
| 3 | Diseases of the blood or blood-forming organs |  |  | [122] | 1 (1%) |
| 4 | Diseases of the immune system |  |  |  |  |
| 5 | Endocrine, nutritional or metabolic diseases | [19, 34, 105, 114, 115] | 5 (11%) | [85, 93, 97, 98, 112, 126] | 6 (8%) |
| 6 | Mental, behavioral or neurodevelopmental disorders | [17, 21, 71, 73, 76, 79, 102, 103, 116, 124] | 10 (22%) | [45, 52, 56, 57, 66, 69, 86, 94, 96, 109] | 10 (14%) |
| 7 | Sleep-wake disorders |  |  | [95] | 1 (1%) |
| 8 | Diseases of the nervous system |  |  | [38, 50, 80, 111] | 4 (6%) |
| 9 | Diseases of the visual system | [72] | 1 (2%) | [64, 65] | 2 (3%) |
| 10 | Diseases of the ear or mastoid process |  |  |  |  |
| 11 | Diseases of the circulatory system | [23, 117, 123, 130] | 4 (9%) | [41, 47, 68, 108, 125, 132] | 6 (8%) |
| 12 | Diseases of the respiratory system | [28, 30] | 2 (4%) | [63, 90, 92, 106, 107, 121] | 6 (8%) |
| 13 | Diseases of the digestive system |  |  |  |  |
| 14 | Diseases of the skin |  |  |  |  |
| 15 | Diseases of the musculoskeletal system or connective tissue | [32, 36, 91] | 3 (7%) |  |  |
| 16 | Diseases of the genitourinary system | [37] | 1 (2%) | [58, 82, 113] | 3 (4%) |
| 17 | Conditions related to sexual health |  |  |  |  |
| 18 | Pregnancy, childbirth or the puerperium |  |  |  |  |
| 19 | Certain conditions originating in the perinatal period |  |  |  |  |
| 20 | Developmental anomalies | [22] | 1 (2%) |  |  |
| 21 | Symptoms, signs or clinical findings, not elsewhere classified | [20, 25, 29, 31, 74, 75, 100, 101] | 8 (17%) | [35, 39, 59, 67, 112, 133] | 6 (8%) |
| 22 | Injury, poisoning or certain other consequences of external causes |  |  | [48, 51, 53, 55, 84, 87, 131] | 7 (10%) |
| 23 | External causes of morbidity or mortality |  |  |  |  |
| 24 | Factors influencing health status or contact with health services |  |  | [83, 89, 110] | 3 (4%) |
| 25 | General patient population | [18, 24, 27, 33, 77, 78, 118, 129] | 8 (17%) | [40, 42, 81, 120, 127, 128] | 6 (8%) |

*Table 2: Overview of the scope problem. Design papers are contrasted to evaluation papers. The table illustrates what other users than patients were considered in the design and evaluation processes and if these users provided input into their own needs, or into patient needs.*

|  | **Design papers**  n=46 | | | **Evaluation papers**  n= 71 | | |
| --- | --- | --- | --- | --- | --- | --- |
|  | ***Input on patient needs*** | ***Input on own needs*** | **Frequency**  n (%) | ***Input on patient needs*** | ***Input on own needs*** | **Frequency**  n (%) |
|  |  |  |  |  |  |  |
| No other users considered | [17, 18, 23, 29, 72, 79, 102, 115, 116, 123, 124] | | 11 (24%) | [39, 41-50, 52-55, 57, 59-70, 80-91, 93-99, 106, 107, 111-113, 120, 121, 125, 126, 131-133] | | 59 (83%) |
| Caregiver | [21, 37, 114, 130] | [20, 24, 26, 30-32, 34, 36, 71, 73-78, 101, 103-105, 117, 119, 129] | 4/22 (9%/48%) | [38, 40, 108, 109, 122, 127, 128] | [56, 110] | 7/2 (10%/3%) |
| Relatives | [22] | [25, 26, 28, 32, 76, 100, 101] | 1/7 (2%/15%) |  | [51, 58, 110] | 0/3 (0%/4%) |
| Technical expert | [19, 20, 27, 71, 104, 105, 129] |  | 7/0 (15%/0%) |  |  |  |
| Expert on health status | [19-21, 24, 27, 28, 33, 37, 74, 75, 101] |  | 11/0 (24%/0%) |  |  |  |
| Government | [27] |  | 1/0 (2%/0%) |  |  |  |
| Visualizing actor network | [35, 118] |  | 2/0 (4%/0%) |  |  |  |

*Table 3: Overview of the scope problem. Only evaluation papers are studied. The table illustrates if wellbeing was considered as the main outcome, the secondary outcome, or an indirect effect in the study. The table also provides insight into what timespan is considered to evaluate wellbeing.*

|  | **Main outcome**  (n=50, 70%) | **Secondary outcome**  (n=11, 15%) | **Indirect effect**  (n=9, 13%) | **Frequency**  n (%) |
| --- | --- | --- | --- | --- |
|  |  |  |  |  |
| Measurement during use period | [40, 48, 82, 86, 111, 113, 122, 128] | [62, 126, 132] | [41, 120] | 8/3/2 (11/4/3 -18%) |
| Measurement direct after use | [39, 46, 47, 50, 52, 54, 55, 57-61, 66, 68-70, 81, 83, 86-89, 92, 94, 96-99, 107, 108, 121] | [67, 80, 84, 90, 109, 110, 112] | [38, 43, 49, 131, 133] | 31/7/5 (44/10/7 – 61%) |
| Measurement 0-1 month after use | [60, 95] |  | [49, 125] | 2/0/2 (3/0/3 – 6%) |
| Measurement 1-3 months after use | [51, 52, 63, 64, 69, 70, 87, 95] | [44, 84, 112] |  | 8/3/0 (11/4/0 - 15%) |
| Measurement 3-6 months after use | [45, 51, 53, 56, 63, 85, 95, 97, 106] |  | [125] | 9/0/1 (13/0/1 - 14%) |
| Measurement 6-12 months after use | [45, 53, 56, 65, 85, 93, 97] | [44, 62] |  | 7/2/0 (10/3/0 - 13%) |
| Other | [127] |  | [42] | 1/0/1 (1/0/1 -2%) |

**Specification problem**

*Table 4: Overview of the specification problem. Design papers are contrasted to evaluation papers. The table illustrates what types of digital health technologies are studied.*

|  | **Design papers**  n=46 | **Frequency**  n (%) | **Evaluation papers**  n=71 | **Frequency**  n (%) |
| --- | --- | --- | --- | --- |
|  |  |  |  |  |
| Supporting platform (app/internet) | [18, 19, 22, 24-28, 32-37, 71, 74-78, 100, 102, 104, 105, 114, 115, 117, 118, 124, 130] | 30 (65%) | [40, 41, 44, 46-48, 51, 52, 54, 55, 58-60, 62, 67-70, 80, 82, 86, 87, 91, 93-97, 106, 108, 110, 112, 121, 122, 126, 127, 131, 132] | 38 (54%) |
| Sensor (also smartphone sensing, ECG sensing, ) | [17, 23, 29-31, 73, 79, 123] | 8 (17%) | [38, 41, 42, 45, 57, 90, 133] | 7 (10%) |
| Telephone or video based support |  |  | [39, 49, 53, 56, 61, 63, 85, 89, 92, 98, 107, 125] | 12 (17%) |
| Social media/internet in general |  |  | [109, 113, 128] | 3 (4%) |
| Audiovisuals/ Serious gaming/ Virtual reality | [116, 119] | 2 (4%) | [43, 50, 83, 84, 88, 99, 111] | 7 (10%) |
| Clothes (goggles/shirt/necklace) | [31, 72, 103] | 3 (7%) | [64, 65] | 2 (3%) |
| Other | [21] | 1 (2%) | [120] | 1 (1%) |
| General | [20, 101, 129] | 3 (7%) | [81] | 1 (1%) |

*Table 5: Overview of the specification problem. Design papers are contrasted to evaluation papers. The table illustrates the most frequent locations of digital health use.*

|  | **Design papers**  n=46 | **Frequency**  n (%) | **Evaluation papers**  n=71 | **Frequency**  n (%) |
| --- | --- | --- | --- | --- |
|  |  |  |  |  |
| Home | [17-23, 25-37, 71-79, 100-105, 114-119, 123, 124, 129, 130] | 45 (98%) | [38-42, 44-63, 66-70, 80-82, 84-87, 89-99, 106-109, 112, 113, 120-122, 125-128, 131-133] | 64 (90%) |
| Home of caregiver |  |  | [56] | 1 (1%) |
| Clinic/ primary care | [24] | 1 (2%) |  |  |
| Hospital | [119] | 1 (2%) | [43, 88] | 2 (3%) |
| Palliative care setting |  |  | [83, 110] | 2 (3%) |
| Public space | [72, 79] | 2 (4%) | [64, 65] | 2 (3%) |

*Table 6: Overview of the specification problem. Only design papers are studied. The table illustrates what design methods are commonly considered to design for wellbeing.*

|  | **Design papers**  n=46 | **Frequency**  n (%) |
| --- | --- | --- |
|  |  |  |
| Interviews | [21, 24, 26-28, 30, 31, 34, 36, 76, 103, 115-118, 129] | 16 (35%) |
| Testing prototype | [19, 22, 24, 28, 30-32, 36, 72, 74, 75, 77, 78, 102, 104] | 15 (33%) |
| Focus group/ workshop | [28, 33, 34, 36, 37, 71, 77, 78, 103, 105, 118, 129, 130] | 13 (28%) |
| Variety of design methods | [17-20, 22, 23, 25-27, 29, 32, 34-36, 71, 73, 79, 100, 101, 103, 114, 116-119, 123, 124, 129] | 28 (61%) |

*Table 7: Overview of the specification problem. Only design papers are studied. The table illustrates how design requirements are constructed.*

|  | **Design papers**  n=46 | **Frequency**  n (%) |
| --- | --- | --- |
|  |  |  |
| User input generally translated into design requirements | [18-21, 24-33, 35, 36, 71-78, 100-105, 115-117, 124, 129, 130] [34, 118] | 39 (85%) |
| Creation by designer | [17, 114, 123] | 4 (8%) |
| Persona translation into design requirements | [103, 119] | 2 (4%) |
| Other | [23, 37, 79] | 4 (9%) |

*Table 8: Overview of the specification problem. Only evaluation papers are studied. The table illustrates what evaluation methods are commonly considered to evaluate digital health for wellbeing.*

|  | **Evaluation papers**  n=71 | **Frequency**  n (%) |
| --- | --- | --- |
|  |  |  |
| Pre-post measurement via questionnaire | [40, 41, 47, 50, 52, 55, 57, 63-66, 69, 85-89, 92, 94, 96, 97, 111, 125] | 23 (32%) |
| Randomized controlled trial (comparison with control group) | [43-45, 49, 51, 53, 56, 59-62, 67, 68, 70, 82, 84, 90, 93, 95, 98, 99, 112] | 22 (31%) |
| Usability - interviews | [38, 39, 46, 54, 55, 80, 83, 106-108, 110, 121, 126, 131, 132] | 15 (21%) |
| Usability - questionnaires | [46, 83, 113] | 3 (4%) |
| Analysis of technology use through data | [42, 48, 91, 122, 133] | 4 (6%) |
| Population survey | [81, 127, 128] | 3 (4%) |
| Partially randomized patient preference design | [58] | 1 (1%) |
| Other | [109, 120] | 2 (3%) |

**Aggregation problem**

***Table 9: Overview of the aggregation problem. Design papers are contrasted to evaluation papers. The table illustrates if value conflicts were studied in the papers, and between which users these value conflicts occurred.***

|  | **Design papers**  n=46 | **Frequency**  n (%) | **Evaluation papers**  n=71 | **Frequency**  n (%) |
| --- | --- | --- | --- | --- |
|  |  |  |  |  |
| No value conflicts | [17-19, 21-26, 28-37, 71, 76-79, 100, 103-105, 114-116, 119, 123, 124, 129] | 35 (76%) | [39-70, 80, 82-88, 90-99, 106-113, 120-122, 125, 126, 128, 131, 132] | 66 (93%) |
| Conflict within one user | [74] | 1 (2%) | [82] | 1 (1%) |
| Conflict within users of same group | [74, 130] | 2 (4%) | [38, 81, 89, 133] | 4 (6%) |
| Conflict between users of different groups | [20, 27, 72-75, 101, 102, 117, 118] | 10 (22%) | [127] | 1 (1%) |

***Table 10: Overview of the aggregation problem. Design papers are contrasted to evaluation papers. The table illustrates what papers considered personalization of digital health.***

|  | **Design papers**  n=46 | **Frequency**  n (%) | **Evaluation papers**  n=71 | **Frequency**  n (%) |
| --- | --- | --- | --- | --- |
|  |  |  |  |  |
| No personalization reported | [19, 24-26, 28-32, 34, 36, 37, 71-73, 79, 102-105, 115, 116, 118, 123, 129, 130] | 26 (57%) | [40, 42, 45-47, 50-55, 61-65, 67-70, 80, 82, 83, 85, 87, 88, 90, 92-94, 96, 97, 106, 109, 121, 125-128, 131] | 40 (56%) |
| Personalization reported | [17, 18, 20-23, 27, 33, 35, 74-78, 100, 101, 114, 117, 119, 124] | 20 (43%) | [38, 39, 41, 43, 44, 48, 49, 56-60, 66, 81, 84, 86, 89, 91, 95, 98, 99, 107, 108, 110-113, 120, 122, 132, 133] | 31 (44%) |
